# Complex Roles of Insect Cytochrome P450s in Chemical Adaptation

**DOI:** 10.7150/ijbs.135523

**Published:** 2026-06-10

**Authors:** Qi-Ren Chen, Timothy W. Moural, Fang Zhu

**Affiliations:** 1 Department of Entomology, Pennsylvania State University, University Park, PA 16802, USA.; 2 Huck Institutes of the Life Sciences, Pennsylvania State University, University Park, PA 16802, USA.

**Keywords:** cytochrome P450, metabolic detoxification, pesticide resistance, cuticular penetration, symbiont-mediated modulation

## Abstract

The remarkable success of insects is largely due to their capacity to adapt to various environmental stresses. Central to their ability to cope with chemical challenges, cytochrome P450 monooxygenases (P450s) form a highly diversified superfamily that enables this capacity through extensive functional and evolutionary plasticity. Here, we examine the diversity of insect P450s, including their evolution, classification, and structural features. We bring together recent advances to highlight the intricate and interconnected roles of P450s, which are repeatedly co-opted across diverse mechanisms of chemical adaptation and extend across conventional clan boundaries. By integrating functional, evolutionary, and structural perspectives, we propose a holistic framework in which insect P450s act as cross-mechanism and cross-clan nodes linking detoxification, cuticular penetration, olfaction, and symbiont-mediated chemical adaptation. This framework provides a systems-level perspective on how P450 diversification shapes insect responses to chemically complex environments.

## Introduction

Insects are continually challenged by a wide variety of chemical compounds arising from both natural and anthropogenic sources, including plant allelochemicals, microbial metabolites, and synthetic insecticides 1-4. Many of these compounds impose strong selective pressures due to their toxic, deterrent, or growth-inhibitory effects, whereas others function as informational cues that mediate interactions among insects, plants, and microbes 5, 6. The ability to tolerate, detoxify, and adapt to these chemical compounds is a fundamental determinant of insect survival, ecological specialization, and evolutionary success 7-9. As insects have radiated into diverse habitats and adopted highly variable feeding strategies, chemical adaptation has become a central theme in insect physiology, biochemistry, ecology, and pest management research 9, 10.

To persist in these chemically complex environments, insects have evolved multiple adaptive strategies, including behavioral avoidance, reduced cuticular penetration, enhanced enzymatic detoxification, symbiont-mediated metabolism, and target-site insensitivity 1, 9, 11-13. Among detoxification enzymes, cytochrome P450 monooxygenases (P450s) are particularly important. They represent one of the most versatile and influential enzyme families involved in chemical adaptation 2, 14, 15. P450s are a large superfamily of heme-thiolate enzymes first discovered in 1958 14. They are ubiquitous across all domains of life, including bacteria, protists, plants, fungi, animals, and even viruses, and play central roles in the metabolism of endogenous compounds and exogenous xenobiotics 16. P450 enzymes exhibit exceptional catalytic versatility because their heme iron center can access multiple electronic and spin states, enabling flexible frontier orbital interactions and supporting multiple reaction pathways 17. Since the first insect cytochrome P450 gene, *CYP6A1*, was cloned and sequenced from the house fly (*Musca domestica*), advances in genome sequencing have revealed an unprecedented level of P450 diversity in insects 14. Genome assemblies are now available for more than 2,600 insect species 18, exposing extensive lineage-specific expansions and functional diversification of P450 genes across the insect tree of life 19. Insect P450s perform essential endogenous functions throughout all life stages, including the biosynthesis and metabolism of hormones (e.g. ecdysteroids and juvenile hormones), lipids, and cuticular hydrocarbons, as well as pheromone degradation 20, 21. At the same time, many insect P450s function as environmentally responsive enzymes that detoxify xenobiotics, including plant secondary metabolites and synthetic insecticides 3, 22. The remarkable functional diversity of insect P450s likely reflects conserved catalytic quantum chemistry combined with an unusually evolvable protein scaffold. This enables repeated co-option for metabolizing diverse xenobiotics and mediating chemical communication 19, 23.

Although many insect P450s have been functionally characterized, the vast majority of identified P450 genes remain functionally unstudied 24. Consequently, our understanding of how P450s act in concert to mediate complex chemical adaptation processes remains limited. Recently, several excellent publications have documented the expansion of P450 gene families, diverse regulatory mechanisms, and broad metabolic capacities across insect taxa 2, 3, 19, 24-27. Building on these foundations, here we focus on the diversity of insect P450s, including their evolution, classification, and structural features. We also synthesize recent advances to highlight the complex and interconnected roles of insect cytochrome P450s, which are often co-opted across multiple mechanisms of chemical adaptation and span traditional P450 clans. Specifically, we emphasize four interrelated aspects: (i) roles of P450s in host-plant adaptation and chemical communication, (ii) P450s contributions to insecticide resistance and metabolism, (iii) cuticle-associated P450s in chemical adaptation, and (iv) symbiont-mediated modulation of P450 detoxification. By integrating functional, evolutionary, and structural perspectives, we propose a holistic framework for insect P450s, including hormone-biosynthetic and detoxification-associated families. In this framework, P450s act as cross-mechanism and cross-clan nodes linking detoxification, cuticular penetration, olfaction, and symbiont-mediated chemical adaptation.

## Evolution of insect P450s

Although the evolutionary origin of the cytochrome P450 superfamily remains unclear, several hypotheses offer insight into the biochemical pressures that have shaped these enzymes 28. Before acquiring the ability to bind oxygen, early P450s may have functioned as reductases in oxygen-free environments or as peroxygenases catalyzing basic oxidative reactions 29, 30. With the rise of atmospheric and cellular oxygen, reactive oxygen species (ROS) posed significant threats to cellular integrity. Early P450 enzymes were recruited to detoxify ROS, mitigating oxidative damage to critical biomolecules 31. The oxygenated products of these reactions, such as sterols and membrane lipids, subsequently assumed important roles as metabolites and signaling molecules, thereby creating new chemical substrates for P450-catalyzed transformations 32, 33. In insects, this ancient versatility was retained and further elaborated. P450 enzymes perform essential roles in the biosynthesis of hormones, pheromones, and other key metabolites that regulate development, reproduction, and communication 14, 34, 35. Subsequent ecological pressures, particularly exposure to plant secondary metabolites and other xenobiotics, promoted the expansion and functional diversification of insect P450s, enhancing their capacity for detoxification and chemical adaptation. Host plant chemistry and dietary breadth exert strong selective pressure on these genes, with generalist insects typically harboring larger and more diversified P450 repertoires than specialists 36-39. At the genomic level, these adaptive responses are largely mediated by gene duplication and gene birth-death dynamics, which provide the raw material for the expansion and diversification of insect P450 families 40, 41. In addition, recent anthropogenic pressures such as extensive insecticide application may further shape the evolution and selection of detoxification-related genes in some insect lineages. For example, comparative genomics of the CYP9A subfamily in the noctuid pests *Spodoptera frugiperda* and *S. exigua* revealed species-specific gene duplications and sequence divergence within large CYP9A clusters 42. Another well characterized case is the evolution of fenvalerate resistance in Australian *Helicoverpa armigera*, where a chimeric P450, CYP337B3 arose through gene duplication and recombination. This case illustrates how P450 gene birth-death dynamics can drive rapid adaptive evolution 43. Additionally, P450 genes are frequently organized in tandem clusters, or near transposable element rich regions, which promote local duplication and rapid turnover through unequal crossing-over and recombination 23, 44, 45. Moreover, regulatory evolution, including changes in inducibility, tissue specificity, and *cis-* or* trans-* regulatory signaling, provides an additional mechanism for rapid adaptation without the need for new genes 25. Compared to vertebrates, insect P450 exhibits rapid evolution by high gene birth-death rates, sequence divergence, and lineage-specific innovation, particularly in environmental sensing, detoxification, and ecological interactions 40. Across insect species, no clear correlation is observed between CYPome size and genome size (Fig. S1). For example, species with relatively large genomes (e.g., *Locusta migratoria* 6.5 Gb; *Periplaneta americana* 3.38 Gb; *Diabrotica virgifera virgifera* 2.42 Gb) exhibit substantial variation in CYPome size: while *L. migratoria* (94) and *D. virgifera virgifera* (105) have average-sized CYPomes, *P. americana* has one of the largest (178). Similarly, species with relatively small genomes (e.g., *Culex quinquefasciatus* 540 Mb, *Tribolium castaneum* 204 Mb) have some of the largest CYPomes (Fig. S1, Table S1). This pattern is consistent with previous studies showing that arthropod CYPomes are shaped by lineage-specific expansions (“bloom”) and dynamic birth-death processes, rather than uniform genome-wide expansion 19.

## Classification and functional diversity of insect P450s

**Figure 1:**
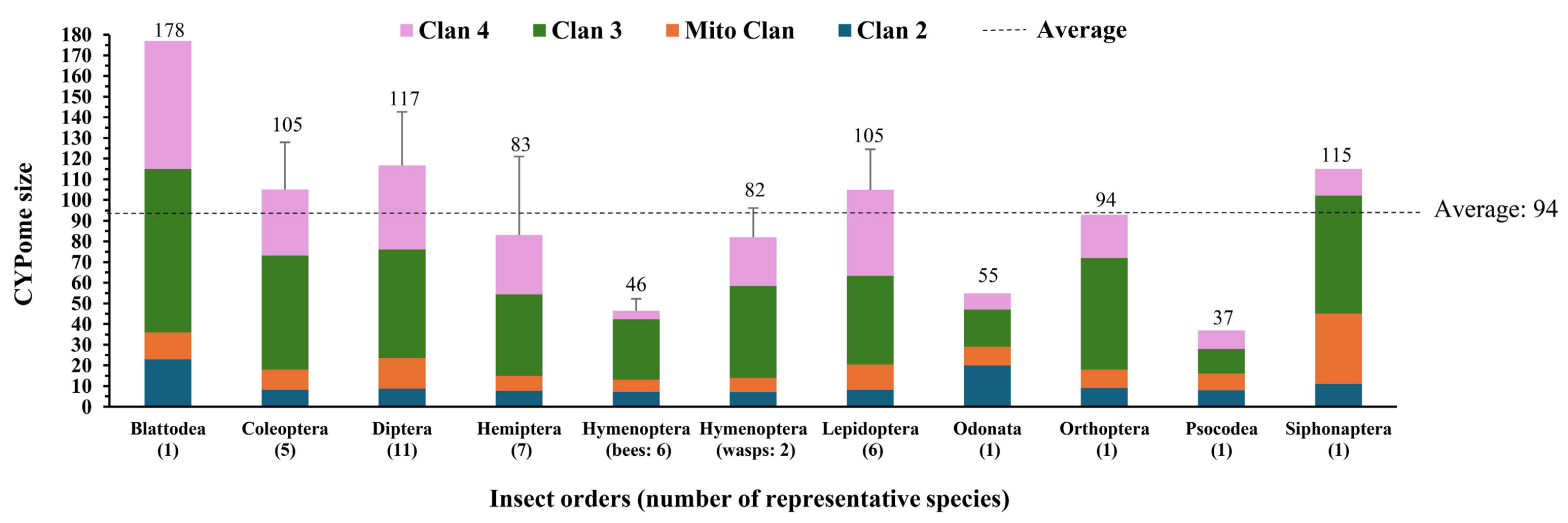
Average cytochrome P450 (CYP) gene numbers across insect orders based on the species listed in Table S1, with bees and wasps shown separately. The y-axis shows CYPome size (number of CYP genes), and the x-axis shows insect orders. Bars represent the mean number of CYP genes per species within each group, categorized into the four major CYP clans (CYP2, mitochondrial, CYP3, and CYP4). The dashed line indicates the average CYPome size across all species analyzed.

Despite extreme sequence diversity, insect CYP genes cluster into four conserved evolutionary clans: CYP3, CYP4, CYP2 and the mitochondrial clan. More recently, additional minor clans (e.g., clans 16 and 20) have been recognized in some lineages, although their functional roles remain largely unknown compared with the major CYP clans 19. Table S1 summarizes CYP gene repertoires from 50 insect species representing major taxonomic groups, including agricultural pests, urban pests, and pollinators. Data were compiled from genomic studies with eight species represented by transcriptome-based datasets. The average insect CYPome size is 94 genes (Fig. 1; Table S1). Differences in CYPome size among insect orders are largely attributable to evolutionary dynamics and functional diversification of the four major CYP clans. The insect CYP3 clan represents the most expanded and diversified clan in insects 14, 40, 46. The CYP3 clan comprises families such as CYP6, CYP9, CYP28, and many CYP300-400 series members and is most closely related evolutionarily to vertebrate CYP3 and CYP5 families. Members of this clan are frequently involved in xenobiotic metabolism, host-plant adaptation, and insecticide resistance, and are often organized in tandem gene clusters with tissue- and stage-specific expression patterns 14, 23, 40, 46. Following duplication, many CYP3 paralogs may experience relaxed purifying selection, permitting sequence divergence and functional differentiation while retaining partial redundancy 47. In *T. castaneum*, detoxification-related P450s, particularly within the CYP3 clans, have undergone extensive lineage-specific expansion. The *T. castaneum* genome encodes a markedly expanded repertoire of CYP3 members (27 subfamilies, 79 genes), especially within the CYP6 and CYP9 families 10.

Long-term exposure to diverse xenobiotics, particularly plant allelochemicals, has likely driven the retention and expansion of CYP3 gene clusters, while more recent exposure to synthetic insecticides has further selected for enhanced metabolic capacity 47, 48. Lepidoptera provides a well-characterized example in which extensive P450 clustering reflects repeated tandem duplication driven by chronic exposure to chemically diverse host plants. Detoxification-related P450s, especially those in the CYP3 clans, evolve under a birth-death model characterized by high duplication and loss rates and relaxed functional constraints, favoring the accumulation of clustered paralogs 7, 42. These clusters are frequently located in transposable element-rich genomic regions, which could facilitate local duplication via unequal crossing-over 44, 45. In addition, physical clustering may confer functional and evolutionary advantages by maintaining groups of detoxification genes with complementary activities, enhancing the ability to cope with diverse allelochemicals and insecticides 49. Variation in CYPome size may also reflect ecological adaptation. Polyphagous insects exposed to diverse host plant allelochemicals and environmental xenobiotics often exhibit expanded detoxification-related CYP families, whereas specialist species may retain more restricted CYP repertoires associated with narrower feeding niches. Mosquitoes (e.g., *C. quinquefasciatus, Aedes aegypti, Anopheles gambiae*) maintain relatively large CYP repertoires, likely reflecting the functional diversity of these enzymes in metabolizing insecticides, environmental pollutants, and host-derived compounds. Such diversity might confer metabolic flexibility and facilitate the rapid evolution of resistance under strong vector-control selection pressure 50. Similarly, the American cockroach (*P. americana*) possesses one of the largest CYPomes reported in insects, primarily due to extensive expansion of the CYP3 clan genes. This expansion may enhance detoxification capacity towards diverse xenobiotics and support adaptation to chemically complex urban and waste-associated habitats, consistent with its highly generalist feeding ecology 51. In contrast, extreme ecological specialization is correlated with CYPome contraction. *Pediculus humanus* (the human body louse) possesses one of the smallest CYP repertoires among insects, with only 12 CYP3 genes, consistent with long-term genome streamlining. As an obligate ectoparasite feeding exclusively on human blood, it encounters relatively limited chemical diversity and reduced exposure to environmental xenobiotics 52.

The CYP4 clan comprises the CYP4 family, which contains members from vertebrates and insects, as well as several families (e.g., CYP311-316) that can be traced back to ancestral genes in *Caenorhabditis elegans* 14. CYP4 clan is highly diverse and typically represents the second-largest group in insect CYPomes 46. For example, the *T. castaneum* genome encodes 47 individual genes belonging to 15 subfamilies in the CYP4 clan, with the CYP4 family being particularly expanded and comprising 27 genes (Fig. 1, Table S1) 10, 40. However, the size of CYP4 clan varies remarkably among insect lineages, and substantial reductions are observed in some taxa. For example, most hymenopteran species harbor fewer than 10 CYP4 genes, while honey bee and other Apoidea only have 3-4 genes in CYP4 clan (Fig. 1, Table S1) 19. Although many members remain poorly characterized, the CYP4 clan exhibits diverse functions in xenobiotic detoxification, cuticular hydrocarbon (CHC) biosynthesis, and fatty acid metabolism 14, 46. Due to their central role in CHC biosynthesis, genes in CYP4G families are conserved across insect lineages with most species possessing at least one *CYP4G* gene 26, 53, 54. For example, honey bee 55 and pea aphid 56 each carry a single *CYP4G* gene in their genomes, whereas Lepidopterans' genomes contain multiple *CYP4G* genes 57.

The mitochondrial CYP clan constitutes a distinct and highly conserved lineage within insect CYPomes. In contrast to plants, which lack mitochondrial P450s, insects and vertebrates retain this clan, although their functional repertoires differ significantly 40. In vertebrates, mitochondrial P450s are primarily involved in steroid and vitamin D metabolism, whereas in insects they play central roles in ecdysteroid biosynthesis, hormone modification, and detoxification 14, 58. As a monophyletic group localized to mitochondria, the mitochondria CYP clan includes highly conserved enzymes that are essential for ecdysteroid biosynthesis, including the Halloween genes CYP302, CYP314, and CYP315 59. While other mitochondrial P450s, particularly in dipterans (e.g. CYP12 family in house fly) are implicated in detoxification, resulting in highest number among other orders 40. Another example is cat flea (*Ctenocephalides felis*), which has expanded CYP12 family in Mitochondria Clan, with 34 genes uncharacterized 60. Recent study showed that some mitochondria P450s such as CYP333B3, CYP305B1, and CYP339A1 in *H. armigera* may also contribute to xenobiotic metabolism 58. Overall, mitochondrial CYP clan size (average ~11 genes) is relatively stable across insect orders, with only modest expansion observed in Diptera and cat flea (Fig. 1, Table S1).

The insect CYP2 clan represents the most ancient P450 lineage and primarily functions in endogenous biosynthesis 46. For example, CYP15, CYP18, and several CYP300-series families, are characterized by strong functional constraints and are implicated in hormone biosynthesis and development-related processes 40, 46. However, recent studies have shown that some CYP2 clan P450s (e.g. CYP306 from *Spodoptera litura*, CYP305B, CYP18A1, and CYP303A1 from *H. armigera*) also participate in xenobiotic detoxification, in addition to their classical endogenous roles 58, 61. The CYP2 clan contains an average of approximately 9 genes across insect orders (Fig. 1, Table S1) and exhibits relatively little inter-order variation compared with the highly dynamic CYP3 and CYP4 clans.

## Structural diversity of insect P450s

Despite the central role of P450s in insect chemical adaptation, high-resolution three-dimensional structures of insect P450s remain scarce 2. To date, most structural insights into P450 enzymes have been derived primarily from crystallographic and cryo-electron microscopy studies of bacterial, plant, and mammalian P450s, particularly human P450s 14, 62, 63. Consequently, structural features of insect P450s are inferred mainly from comparative sequence analysis, homology modeling, and functional studies based on non-insect P450 structures. Nevertheless, the P450 superfamily is evolutionarily conserved, with core catalytic architectures shared across taxa, providing a solid framework for understanding the structure-function relationships of insect P450s involved in chemical adaptation 14, 62, 64.

**Figure 2:**
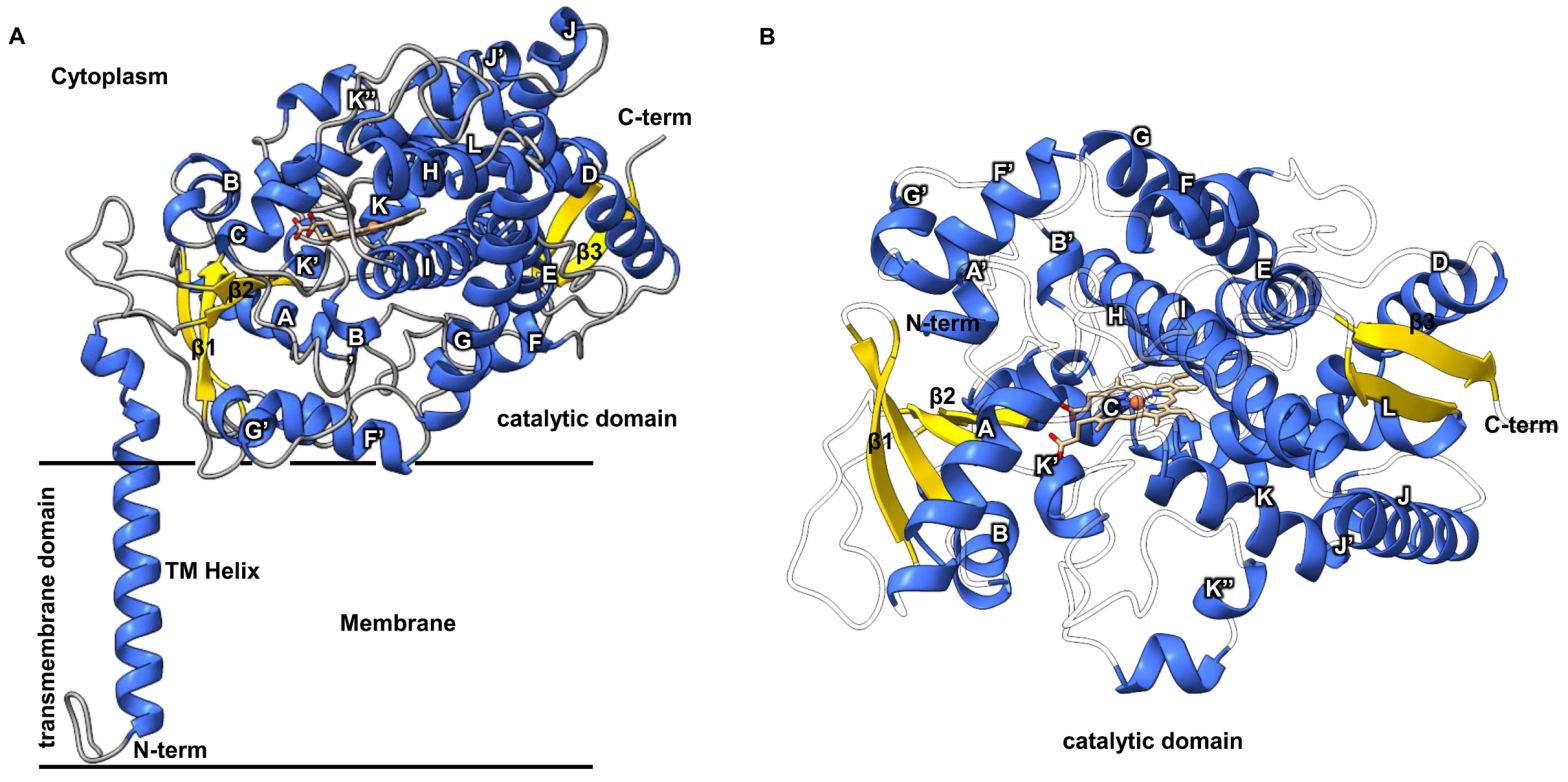
Structural model of an insect P450. The full-length amino acid sequence of CYP6BQ9 from the red flour beetle, *Tribolium castaneum* was used to generate an Alphafold 3 model 199. CYP6BQ9 orientation within the endoplasmic reticulum (ER) was predicted using the PPM 3.0 server 200. **(A)** Structural model of CYP6BQ9 shown in ribbon representation, with α-helices colored blue, β-strands gold, and loops grey. The N-terminal transmembrane (TM) helix is embedded in the lipid bilayer, and the catalytic domain extends into the cytosol. The heme cofactor is shown with carbon atoms colored tan and heteroatoms colored according to the CPK element scheme. Black horizontal lines indicate the membrane boundaries. Secondary structural elements are labeled. **(B)** Catalytic domain of the CYP6BQ9 model, colored and labeled as described in (A). Loops are rendered transparent to provide a clearer view of the heme center.

In general, insect P450s exhibit a conserved global fold, comprising an N-terminal transmembrane domain linked to a larger catalytic domain that contains the heme-binding site responsible for catalysis (Fig. 2A) 65-68. The catalytic domain adopts the classic P450 fold, characterized by a predominantly α-helical (~12 to 16 helices) with three to four β-sheets, and is organized into two sub-domains: the β domain and the α domain 65-70. The secondary structural elements of the catalytic domain form a structural scaffold that supports both the active site and centrally located heme cofactor. In P450 structural studies, the hydrophobic transmembrane domain poses challenges for protein crystallization; consequently, most resolved structures lack the N-terminal transmembrane helix (TM helix) and focus on the soluble catalytic domain 71. A notable exception is the full-length CYP51 class P450 from *Saccharomyces cerevisiae* solved by Monke et al. 71. The *S. cerevisiae* CYP51A1 full-length structure provided evidence that the TM helix anchors P450 at the membrane without disrupting the canonical fold of the catalytic domain. The TM-helix spans the membrane, anchoring eukaryotic P450s to the endoplasmic reticulum. The catalytic domain faces the cytosol and interacts with substrates and redox partners, including cytochrome P450 reductase and cytochrome b5 72-74. P450 enzymes are membrane-anchored through the TM-helix, whereas the catalytic domain is only partially embedded in the lipid bilayer via hydrophobic regions such as the F/G loop and are largely exposed to the cytosol (Fig. 2A). This arrangement places the substrate access channel near the membrane interface, facilitating access to both cytosolic and lipophilic substrates 74, 75.

Insect P450s, including members of the CYP6, CYP9, and CYP4 families, are predicted to conform to the canonical P450 structure (Fig. 2A and B) 14, 76. The structural core is formed by the conserved four helix bundle of D, E, I, and L. Leading into helix L is the heme-binding loop with the conserved PFxxGxRxCxG/A motif and the conserved cysteine that acts as the fifth ligand to the heme cofactor's iron, and a defining feature of all P450s 65, 69, 77. This motif is conserved across insect P450 families, including detoxification-associated CYP6 and CYP9 enzymes, suggesting that their catalytic mechanisms resemble those of other eukaryotic and prokaryotic systems. There are five conserved P450 motifs that play central roles in structural fold stabilization, heme binding and/or catalysis. Besides PFxxGxRxCxG/A, the other motifs are WxxxR, GxE/DTT/S, ExxR, and PxxFxPE/DRF 14, 64, 78. The WxxR motif is located on helix C, where the R residue binds one of the heme propionate groups. The GxE/DTT/S motif, located in the middle of helix I adjacent to the heme, contains a catalytically conserved threonine 64. The ExxR motif, located in helix K, establishes a salt bridge network that stabilizes the overall protein structure 78. Lastly, the proline-enriched PxxFxPE/DRF motif, located between the TM-helix and helix A, is thought to contribute to membrane positioning 78.

In contrast to the highly conserved heme-thiolate core, insect P450s exhibit substantial structural diversity within substrate recognition sites (SRSs), which are key determinants of substrate specificity and catalytic versatility. The six canonical SRS regions (SRS1-SRS6), primarily located in flexible structural elements such as the B′ helix, F-G region, and surrounding loops, display significant sequence and conformational variability among insect P450s 14, 79. This structural plasticity enables the formation of diverse substrate-binding cavities and access channels, allowing insect P450s to accommodate a wide range of chemically distinct compounds, including plant allelochemicals and synthetic insecticides. Such diversity in SRS architecture is thought to underlie the remarkable adaptive capacity of insect P450s in response to environmental chemical challenges 80. Comparative analyses indicate that SRS regions are among the most variable regions in insect P450s, particularly within expanded detoxification-related families such as CYP6 and CYP9. For example, CYP6 enzymes involved in pyrethroid or neonicotinoid metabolism often display marked sequence divergence in predicted SRS regions, consistent with differences in substrate specificity and catalytic efficiency 81. The plasticity of the active-site cavity is a defining feature of P450 enzymes and plays a crucial role in insect chemical adaptation. Structural studies of mammalian P450s have shown that even modest amino acid substitutions within SRSs can significantly alter active-site volume, shape, and physicochemical properties 82, 83. Amino acid substitutions within the SRS regions may alter substrate accessibility to the binding pocket or the range and catalytic efficiency of substrate metabolism, ultimately modulating the metabolic capacity of cytochrome P450 enzymes 80. Although direct structural data for insect P450s remain limited, homology models of CYP6 and CYP9 enzymes support the hypothesis that adaptive changes in these regions facilitate the metabolism of chemically diverse compounds, including plant allelochemicals and synthetic insecticides 80, 84. *In vitro* domain-swapping experiments involving SRS1 and SRS6 between CYP6AE17 and CYP6AE18 resulted in reciprocal changes in esfenvalerate-metabolizing activity. In addition, reciprocal exchange of the SRS1 regions between CYP6AE11 and CYP6AE20 caused improper folding of CYP6AE11/20 chimera, whereas the CYP6AE20/11 chimera acquired moderate esfenvalerate-metabolizing activity. These findings demonstrate that sequence variation within SRSs can influence protein folding, active-site conformation, and catalytic properties, thus contributing to the functional divergence of P450 enzymes 85. In several insect species, amino acid substitutions within the predicted substrate-binding regions of P450 proteins (particularly CYP6) have been associated with insecticide resistance, illustrating how active-site plasticity enables adaptive detoxification 86, 87.

## Functional versatility of insect P450s in chemical adaptation

**Figure 3:**
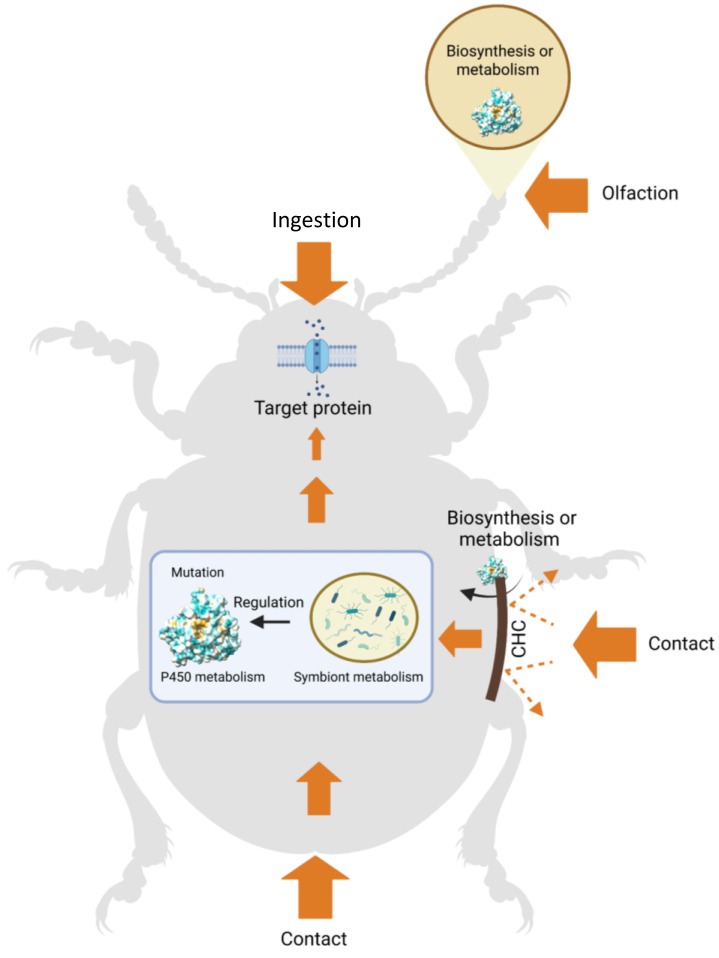
Schematic overview of the diverse functions of insect cytochrome P450s and the evolutionary mechanisms underlying xenobiotic adaptation. Orange arrows indicate the direction and relative magnitude of chemical flux. Chemicals enter insects through multiple routes, including ingestion, antennal pores, cuticular penetration, and anal or ovipositional openings. The cuticle serves as a primary physical barrier to chemical entry, although cuticle-associated P450s may participate in the metabolism or biosynthesis of certain compounds. Chemicals entering through the antennae may also be metabolized by local P450s involved in olfactory processing. Compounds entering via ingestion, cuticular penetration, antennal exposure, or reproductive and anal routes may be metabolized by host P450s or symbiotic microorganisms before reaching their target sites. In addition, microbial symbionts may modulate host P450 expression and influence detoxification capacity. Figure created using BioRender.com.

Cytochrome P450s are one of the most ancient and expansive enzyme superfamilies, known for catalyzing a wide range of oxidative transformations 23. These enzymes mediate diverse reactions, including hydroxylation, epoxidation, and various dealkylation and oxidation processes involving oxygen, nitrogen, and sulfur atoms 14. Their exceptional catalytic plasticity and broad substrate specificity enable insects to metabolize diverse endogenous and exogenous compounds 36. As a result, P450s are key players in the detoxification and metabolic adaptation of insects to both naturally occurring toxins and synthetic xenobiotics, such as pesticides 22, 88. In this review, we highlight the multifaceted roles of insect P450s in four major contexts of chemical adaptation: (1) P450-mediated host-plant adaptation and chemical communication, (2) P450-mediated detoxification of insecticides, (3) cuticle-associated resistance mechanisms, and (4) symbiont modulation of P450-mediated detoxification (Fig. 3).

### Roles of P450s in host-plant adaptation and chemical communication

Insects and plants have interacted for hundreds of millions of years, influencing each other's evolution through long-term ecological and chemical relationships 9, 89. As the primary food source for herbivorous insects, plants have evolved a diverse arsenal of allelochemicals to deter herbivory 36, 37. These compounds include a wide range of insect repellents and toxins, such as alkaloids, terpenoids, phenolics, glucosinolates, cyanogenic glycosides, and proteinase inhibitors 12, 36, 90, 91. In addition to direct-acting allelochemicals, plants employ indirect defenses through the release of herbivore-induced plant volatiles (HIPVs). Although chemically diverse, HIPVs primarily act as airborne signals that attract natural enemies of herbivores or prime defenses in nearby tissues and neighboring plants 92. In response, herbivorous insects have developed diverse adaptive strategies to overcome plant chemical defenses, including behavioral avoidance, excretion, sequestration, enhanced metabolic detoxification, and target site insensitivity 9. Among these mechanisms, metabolic detoxification by a variety of enzymes, such as P450s, glutathione S-transferases (GSTs), carboxylesterases (CCEs), and UDP-glycosyltransferases (UGTs), plays a central role in the neutralizing or metabolizing toxic compounds 22. P450s are particularly critical among these enzymes due to their catalytic versatility, broad substrate specificity, and central role in initiating Phase I detoxification reactions 14, 46. Plant allelochemicals are recognized as major evolutionary drivers of P450 diversification in phytophagous insects 9, 93-95. In certain butterfly species, P450 duplications have enabled the detoxification of novel plant allelochemicals, facilitating host-plant shifts and potentially driving ecological divergence and speciation 95.

The evolutionary pressures driven by plant chemical defenses have also shaped different detoxification strategies among herbivorous insects with distinct feeding niches. Herbivorous insects are generally categorized as generalists, which feed on one or a few related plant taxa, or generalists, which consume a wide variety of plants 96. This distinction often correlates with differences in detoxification capacity: specialists tend to evolve highly efficient P450s fine-tuned for a narrow set of allelochemicals, whereas generalists benefit from P450s with broader substrate specificities. For instance, the parsnip webworm (*Depressaria pastinacella*), a specialist feeding on furanocoumarin-rich Apiaceae plants, expresses the P450 enzyme CYP6AB3 97. Its variant, CYP6AB3v2, efficiently metabolizes imperatorin and myristicin, major host plant allelochemicals, highlighting the evolution of highly specialized detoxification mechanisms in specialist herbivores 98, 99. In contrast, *Helicoverpa zea*, a highly polyphagous generalist, expresses *CYP6B8*, which is induced by allelochemicals such as xanthotoxin and exhibits broad substrate specificity, metabolizing diverse compounds including furanocoumarins and flavonoids 22, 100, 101. Similarly, in the cotton bollworm, *H. armigera*, expansion of the CYP6AE subfamily enhances the capacity to metabolize diverse plant allelochemicals, contributing to its broad host range and dietary plasticity 94. Functional divergence within P450 subfamilies may also contribute to species divergence. In a recent study, both the beet armyworm (*S. exigua*) and the fall armyworm (*S. frugiperda*) were shown to possess *CYP9A* subfamily members involved in allelochemical detoxification. CRISPR-mediated knockout of *CYP9A* genes in both species increases susceptibility to imperatorin and xanthotoxin, with species-specific differences in effect size and paralog involvement. These findings highlight lineage-specific functional divergence within the *CYP9A* subfamily that underlies adaptation to distinct host plant chemicals 42.

Importantly, P450-mediated adaptation to plant-derived toxins is not limited to herbivorous pests. Similar mechanisms are observed in pollinators such as the honey bee (*Apis mellifera*), which encounters a wide array of allelochemicals in its nectar, pollen, and propolis, and expresses multiple P450s (e.g. CYP6AS, CYP9Q, CYP336A1) capable of metabolizing flavonoids, alkaloids, and other plant secondary metabolites 102-104. Notably, *CYP4G11* and *CYP9Q1-3* show task- and tissue-specific expression in honeybees. *CYP4G11* is enriched in the antennae and prothoracic and mesothoracic legs of foragers, suggesting a role in chemosensory perception, whereas *CYP9Q1-3* are most highly expressed in the metathoracic legs of foragers, supporting a role in detoxifying pollen phytochemicals 105.

In addition to their role in metabolizing plant toxins, insect P450s are increasingly recognized as odorant degrading enzymes in olfactory tissues, where they degrade volatile compounds and help reset chemoreception 106, 107. Antennae are critical chemosensory organs responsible for detection and processing of environmental chemical cues. In this context, antennal P450s function to maintain chemosensory fidelity by rapidly degrading odorant molecules, thus preventing receptor overstimulation and ensuring signal clarity 107. A striking example is the mountain pine beetle (*Dendroctonus ponderosae*), which encounters high concentrations of host-derived terpenoids 106. In this species, P450s display remarkable tissue-specific expression patterns in response to chemically complex environments 108. Additionally, cytochrome P450 reductase, the essential electron donor for all P450 genes, is also highly expressed in antennae of Colorado potato beetle and the common bed bug, suggesting that P450-mediated odorant degradation plays a significant role in olfactory tissues 109, 110. P450s expressed in the gut, fat body, and/or Malpighian tubules primarily mediate xenobiotic detoxification, whereas antennal P450s may play multifunctional roles in olfaction, detoxification, and chemical communication 106, 107, 111. For instance, the antenna-specific P450, CYP345E2, in the mountain pine beetle, *D. ponderosae*, metabolizes multiple monoterpenes, including (+)-3-carene, (±)-β-pinene, and (±)-α-pinene, suggesting its dual roles in odorant degradation and protection against the toxic effects of these volatiles 112, 113. Interestingly, antennal P450s may also contribute to pheromone biosynthesis or degradation, highlighting their roles in chemical communication 108. In the mountain pine beetles, *CYP6DE1* is highly expressed in the antennae, heads, and fat bodies and *in vitro* assays show that CYP6DE1 catalyzes the conversion of α-pinene into trans-verbenol, an aggregation pheromone 114. Similarly, in the red imported fire ant (*Solenopsis invicta*), *CYP6K1* and *CYP4V2* are highly expressed in adult worker antennae and are required for the detection of the alarm pheromone 2-ethyl-3,6(5)-dimethylpyrazine (EDMP). Silencing these genes via RNA interference reduces antennal electrophysiological responses and alters behavioral responses to EDMP, indicating that these P450s play critical roles in pheromone perception and signal processing 115. Together, these examples illustrate the multifunctional roles of antennal P450s in insect olfaction, host-plant adaptation, and pheromone-mediated communication.

The evolutionary history of P450-mediated detoxification of plant chemicals may provide important insight into the origins and mechanisms of insecticide resistance. It has been proposed that some insects recruit enzymatic detoxification systems originally evolved for processing plant allelochemicals to metabolize synthetic insecticides 12, 116, 117. This phenomenon, often referred to as 'pre-adaptation', provides a useful framework for understanding cross-resistance 9. Indeed, the long evolutionary history of coping with plant secondary metabolites is thought to contribute to the rapid development of insecticide resistance in some species, mediated in part by shared detoxification pathways involving cytochrome P450s, GSTs, UGTs, and CCEs 118-121. This hypothesis is further supported by the chemical similarities between certain plant-derived compounds and synthetic insecticides, many of which were developed based on natural plant toxins (e.g., pyrethrins and neonicotinoids) 9, 12, 122. In addition, transcriptomic studies have revealed substantial overlap in gene expression responses to plant allelochemicals and synthetic insecticides, particularly in the up regulation of cytochrome P450s and other detoxification enzymes 120, 121, 123. This convergence in detoxification machinery highlights an evolutionary continuum from adaptation to host plant chemistry to contemporary resistance toward synthetic pesticides.

### Roles of P450s contribute to insecticide resistance and metabolism

P450s are among the most important enzymatic systems involved in the metabolism and detoxification of synthetic insecticides and play a central role in the development of insecticide resistance in disease vectors, agricultural and forest pests 3, 124, 125. In addition, P450-mediated detoxification pathways contribute to chemical adaptation in non-target organisms, including both social and solitary pollinators 2, 14.

In mosquitoes, cytochrome P450s are major contributors to resistance against pyrethroids, which have been widely used in indoor residual spraying and long-lasting insecticidal nets 125. Multiple resistance mechanisms have been identified in mosquitoes, with P450-mediated metabolic detoxification being among the most important 50, 126-129. Overexpression of P450s involved in xenobiotic metabolism can arise through several mechanisms, including gene amplification at the genomic level and transcriptional upregulation in resistant strains 2, 128. At the genomic level, copy number variance (CNV) is a common mechanism that increases the number of P450 gene copies, thereby enhancing detoxification capacity. For instance, CNVs in the *Cyp6aa1-Cyp6p2* gene cluster have been documented in *Anopheles* species across Africa, conferring differential levels of resistance to pyrethroids and DDT compared with susceptible strains 130. Beyond gene amplification, overexpression of P450 genes at the mRNA level can also be driven by *cis*- and/or *trans*- acting regulatory changes, including mutations in promoter regions and alterations in transcription factors 2, 131, 132. Several key signaling pathways regulate insect P450 gene expression, including Cap'n'collar C/Nuclear factor erythroid 2-related factor 2-small Maf proteins (CncC/Nrf2-Maf) pathway, the G protein-coupled receptor (GPCR) pathway, and mitogen-activated protein kinase- cAMP responsive element-binding protein (MAPK-CREB) pathway 25. In *An. funestus*, a 3-bp (AAC) deletion in the promoter region of *CYP6P9b*, located 50 bp upstream of a CncC/Nrf2-Maf binding site, has been associated with increased gene expression and pyrethroid resistance in southern African populations 128, 133, 134. In *C. quinquefasciatus*, the GPCR signaling pathway has been implicated in P450-mediated permethrin resistance. Knockdown of four GPCR-related genes resulted in reduced expression of *CYP9M10*, *CYP9J34*, *CYP6AA7*, and *CYP9J40* in a permethrin resistant strain, demonstrating the role of GPCR signaling in the regulation of P450 genes 135. In addition to regulatory changes, recent studies have shown that point mutations in P450 coding sequences can directly alter enzyme activity and enhance catalytic efficiency. In *An. funestus*, site-directed mutagenesis revealed that three amino acid substitutions in CYP6P9b (Val109Ile, Asp335Glu, and Asn384Ser) are key resistance mutations responsible for enhanced pyrethroid metabolism in resistant alleles 136. Similarly, a G454A mutation in CYP9K1 has been shown to increase metabolic activity toward the type II pyrethroid deltamethrin and contribute to elevated resistance in *An. funestus* populations 136, 137. Together, these studies highlight how single or multiple amino acid substitutions in P450 enzymes can significantly alter catalytic efficiency and drive the evolution of insecticide resistance.

P450-mediated detoxification also plays a central role in the development of insecticide resistance in agricultural and forest pests. In the diamondback moth, *Plutella xylostella* (L.), a globally distributed pest of cruciferous crops, functional studies have shown that *CYP6BG1* overexpression may contribute to chlorantraniliprole resistance 138. Similarly, the Colorado potato beetle, *Leptinotarsa decemlineata*, relies heavily on P450-mediated detoxification as a key mechanism of imidacloprid resistance. Numerous P450s in the *CYP4*, *CYP6*, and *CYP9* families are upregulated in resistant strains and can be induced by both host plant allelochemicals and insecticides 121. The CncC/Nrf2-Maf signaling pathway has been identified controlling resistance-associated P450 expression in *L. decemlineata* and *T. castaneum* 139-141. Additionally, microRNA (miRNA)-mediated regulation of P450 detoxification has been identified recently in whiteflies (*Bemisia tabaci*) and planthoppers (*Nilaparvata lugens*), providing new insights into the regulatory mechanisms underlying xenobiotic detoxification 142, 143. In the forest pest *Lymantria dispar*, the methuselah-like *GPCR* gene (*LdMthl1*) and ocular albinism type 1 gene (*LdOA1*) regulate downstream *CYP6* P450 genes, which are involved in deltamethrin resistance 144, 145. Beyond gene amplification and regulatory mutations, emerging evidence suggests that epitranscriptomic regulation may also influence P450 expression and contribute to insecticide resistance. Chemical modifications of mRNA, such as N6-methyladenosine (m⁶A), have been implicated in modulating mRNA stability and translation efficiency, thereby potentially affecting detoxification capacity in insects. Two recent studies showed that m⁶A modification regulates the expression of *CYP4C64* and *CYP417B1*, conferring resistance to thiamethoxam and imidacloprid, respectively 146, 147. In addition, point mutations within the coding regions of P450 enzymes can also contribute to insecticide resistance by altering catalytic efficiency or substrate affinity. For example, an F116I mutation in CYP9A25 of *Spodoptera litura* has been identified as a key determinant of emamectin benzoate metabolism. This mutation, located within substrate recognition site 1 (SRS1), likely enhances metabolic activity by reducing steric hindrance and facilitating substrate access to the enzyme's active site 148. Similarly, amino acid substitutions within the SRS regions of CYP6ER1 in the brown planthopper (*N. lugens*) have been shown to confer resistance to imidacloprid 87.

While cytochrome P450s often contribute to insecticide resistance in pests, they also play crucial roles in detoxifying agrochemicals in pollinators, helping to maintain pollinator health in pesticide-exposed environments 2, 149, 150. Honey bees, once thought to be uniquely sensitive to pesticides due to relatively small repertoire of P450 genes 151, are now recognized as not necessarily among the most sensitive insect species 152, 153. In fact, honey bees are generally more tolerant to agrichemicals, including neonicotinoids and fungicides, than many solitary bee species 154-156. Several CYP9 enzymes have been functionally characterized for their detoxification roles in bees 2. Notably, the CYP9Q family, including CYP9Q1-3 in honey bees and CYP9Q4/5 in bumblebees, effectively metabolizes the neonicotinoid thiacloprid 153. Recent evidence suggests that CYP9Q2 has also shown to metabolize coumaphos, an organophosphate used to manage varroa mites in hives 157. Moreover, CYP9Q2 and CYP9Q3 are involved in the detoxification of insecticides across a diverse range of chemical class, including diamide insecticide chlorantraniliprole and triazole fungicides 158. However, P450-mediated insecticide detoxification capacity varies across bee lineages. In Megachilidae bees, species within the tribes Osmiini and Dioxyini possess *CYP9BU* genes capable of metabolizing thiacloprid 159, whereas the alfalfa leafcutter bee (*Megachile rotundata*) lacks *CYP9Q*-related P450s and substantially more sensitive to certain neonicotinoids 159, 160. Detoxification in bees is a complex process involving not only P450s but also other enzymes, including GSTs, CCEs, UGTs, and ATP-binding cassette (ABC) transporters, as well as the microbiome, all of which may contribute to their adaptation to chemically intensive environments 150, 161. Substantial future research is needed to fully understand the spectrum of genetic and symbiotic factors involved in this process.

### Cuticle-associated P450s in chemical adaptation

The insect integument, composed sequentially from the innermost to outmost layer of the epidermis, endocuticle, exocuticle, and epicuticle, serves as the primary interface between the insect and its external environment. The outermost cuticular layer is coated with cuticular hydrocarbons (CHC), which plays a crucial role in protecting terrestrial insects from desiccation, and acting as signaling molecules in mating and communication 21, 162-165. Accumulating evidence also indicates that CHCs contribute to insecticide resistance by reducing insecticide penetration 4, 26. CYP4G enzymes catalyze the final step of CHC biosynthesis, converting long-chain fatty acyl-CoAs into hydrocarbons via alcohol and aldehyde intermediates 53. In the lower termite *Cryptotermes secundus*, CHCs serve as queen pheromones. RNA interference (RNAi) silencing the *CYP4C1* in queens significantly altered the royal CHC profile, leading to the loss of queen-specific scent and abolished queen recognition by workers 166. While CYP4C1 is distinct from CYP4G enzymes, this study highlights the broader importance of cuticle-associated P450s in shaping CHC profiles that mediate chemical communication, a role that is mechanistically consistent with CYP4G-dependent hydrocarbon biosynthesis 26. Additionally, *Cyp301a1*, a conserved P450 gene belonging to mitochondria clan, is involved in cuticle formation in insects. Although the signaling pathway is not clear, disrupting *Cyp301a1* in *D. melanogaster* results in malformed abdominal cuticles 167.

Given the central role of CYP4G enzymes in CHC production and cuticle integrity, researchers have increasingly investigated their potential contributions to insecticide resistance. The first associations between CYP4G genes and insecticide resistance were reported in *Blattella germanica* insecticide-resistant strains 168. This initial correlative evidence was subsequently supported by functional studies confirming a direct role for CYP4G genes in mediating resistance. In pyrethroid-resistant *An. gambiae*, for instance, overexpression of *CYP4G16* contributes to thicker cuticular hydrocarbon layer compared with susceptible strain. Functional analyses demonstrated that *CYP4G16*, which is highly expressed in oenocytes, the primary cells responsible for CHC production, catalyzes the conversion of long-chain aldehydes into hydrocarbons, confirming its role as a hydrocarbon-forming decarbonylase 169. Similarly, in pyrethroid-resistant *B. germanica*, elevated expression of *CYP4G19* is associated with a thicker CHC layer and reduced cuticular permeability compared with susceptible strain. Conversely, RNAi -mediated knockdown of *CYP4G19* increases the cuticle permeability and insecticide-induced mortality 170. In *N. lugens*, RNAi-mediated silence of *CYP4G76* and *CYP4G115* decreases cuticular hydrocarbon thickness and desiccation tolerance in nymphs while enhancing penetration of pymetrozine, imidacloprid, thiamethoxam, and buprofezin 171.

Although most studies attribute CYP4G-associtated resistance to reduced insecticide penetration through enhanced CHC production, emerging evidence suggests that some cuticle-associated P450s may also contribute to insecticide resistance through direct metabolic detoxification. Evidence from *Liriomyza trifolii* suggests a potential direct metabolic role for CYP4G enzymes. Expression of *CYP4G1* in *Escherichia coli* increased bacterial tolerance to abamectin in survival assays, although further biochemical characterization is needed to confirm direct xenobiotic metabolism by *CYP4G* enzymes 172. Additional support for this possibility comes from studies in the bed bug, *Cimex lectularius*, where many detoxification genes, including several P450 genes highly expressed in pyrethroid-resistant strains, are predominantly expressed in the cuticle. This observation suggests that cuticle-associated detoxification may contribute to resistance 162.

### Symbiont-mediated modulation of P450 detoxification

Microorganisms play a critical role in enabling insects to adapt to diverse environments, including natural, agricultural, and urban environments, reflecting highly dynamic host microbiome interactions 173-175. To survive in chemically complex environments, insects rely on gut associated microbiomes contributing to digestion of polysaccharide, nutrient recycling, and xenobiotic detoxification 174, 175. Although detoxification associated symbionts may not be essential for host growth or reproduction, they are crucial for facilitating chemical adaptation in insects 176.

One of the most direct ways in which symbionts contribute to chemical adaptation is through their ability to metabolize xenobiotics. Numerous studies have demonstrated that gut microbiomes can directly metabolize both plant toxins and synthetic pesticides 1, 177, 178. For example, *Pantoea* spp., harbored by the cabbage stem flea beetle (*Psylliodes chrysocephala*), can degrade plant-derived toxic isothiocyanates *in vitro*. When beetles were treated with antibiotic, the isothiocyanate-degrading capacity was lost but recovered upon reintroduction of *Pantoea*, indicating that these symbionts contribute to host plant toxin detoxification 179. Similarly, in Japanese sugarcane fields with long-term fenitrothion application, a fenitrothion-degrading microbiome, *Burkholderia*, was identified. Bean bug (*Riptortus pedestris*) populations harboring this symbiont showed significantly higher survival than those carrying non-degrading strains, demonstrating direct evidence that *Burkholderia* can confer pesticide resistance 176, 180. Another example occurs in *Grapholita molesta*, where antibiotics-induced dysbiosis of the gut microbiota significantly increased larval sensitivity to emamectin benzoate. In addition, microbiota composition shifted when larvae moved from shoots to fruits, suggesting that dynamic microbiota communities modulate insecticide tolerance in response to dietary changes 181. Together, these studies demonstrate that microbial symbionts can provide an immediate, metabolism-based mechanism for xenobiotic tolerance.

Beyond directly degrading xenobiotics, symbionts can also modulate host detoxification capacity by regulating endogenous detoxification systems, particularly cytochrome P450 enzymes 175, 177. In *A. mellifera*, the gut microbiota promotes the expression of midgut P450s expression, and microbiome-deficient workers show significantly increased susceptibility to thiacloprid and tau-fluvalinate 182. In mosquitoes, antibiotic treatment of* Ae. aegypti* larvae reduced P450 activity. In contrast, artificially increasing the abundance of the gut symbiont *Serratia oryzae* in *Aedes albopictus* larvae elevates P450 and other detoxification enzyme activities, thus improving survival following deltamethrin exposure 183, 184. Similar patterns have been observed in planthoppers (*N. lugens*), where antibiotic treatment downregulated detoxification genes, including *CYP4CE1* and *CYP6ER1*, and increased susceptibility to imidacloprid, chlorpyrifos, and clothianidin 185, 186. However, symbionts do not universally enhance resistance. The symbiont *Arsenophonus* (S-type) reduced expression of *CYP6AY1* and negatively affected imidacloprid resistance compared with the R-type strain, indicating that symbionts can also suppress host detoxification capacity. Such indirect modulation of host enzymatic system is associated with the downregulation of xenobiotic metabolism pathways 187.

In addition to direct and indirect detoxification mechanisms, hosts and their symbionts can cooperate to form integrated detoxification systems. Recent studies described a reciprocal host-symbiont detoxification strategy in which metabolic burdens are partitioned between partners 188. In the bean bug (*R. pedestris*), the gut symbiont *Burkholderia* metabolized organophosphate insecticides into intermediate products. While the parent compound primarily affected the host insect, its metabolite, 3-methyl-4-nitrophenol (3M4N), was lethal to the symbiont. This conflict is resolved through rapid host-mediated excretion of the metabolite, thereby protecting the symbiont and maintaining detoxification capacity 188. Such coordinated metabolic integration highlights how host physiological processes can complement symbiont-mediated xenobiotic metabolism, collectively enhancing pesticide tolerance.

Despite growing evidence for symbiont-mediated detoxification, the molecular mechanisms underlying how symbionts regulate host detoxification pathways in insects remain poorly understood. Nevertheless, insights from vertebrate systems may provide useful conceptual frameworks. RNA-seq comparisons between germ-free and conventionally reared mice have identified differential expression of cytochrome P450 enzymes and associated transcriptional regulators involved in drug metabolism 189, 190. Microbial status and antibiotic exposure (i.e. metronidazole) can modulate transcription factors such as pregnane X receptor (PXR) and Nrf2, providing a mechanistic basis for differential cytochrome P450 expression 191, 192. These findings suggest that microbial metabolites can modulate host signaling pathways that control cytochrome P450 expression. Future studies in insects should therefore focus on key transcriptional regulators and signaling pathways, including GPCRs, MAPK-CREB, AhR/ARNT, HR96, and CncC/Keap1, to elucidate how symbionts influence host detoxification processes 25.

While contributing to short-term physiological modulation, symbionts may also shape the long-term evolutionary trajectory of insect detoxification systems. Growing evidence suggests that microbial genes, including those encoding plant cell wall-degrading enzymes, have been horizontally transferred into insect genomes, representing an additional route for acquiring xenobiotic-degrading capabilities 193. This challenges the traditional view that insect detoxification evolves exclusively through duplication and diversification of endogenous P450 genes 47. Taken together, symbiont-mediated detoxification expands the functional and evolutionary landscape of insect P450-mediated chemical adaptation by integrating microbial metabolism, host regulatory plasticity, and genomic innovation.

## Conclusion and future perspectives

Insect cytochrome P450s play central roles in chemical adaptation by facilitating insects to cope with diverse environmental challenges. Over the past 15 years, research on insect P450s has expanded rapidly, driven by advances in genome sequencing and annotation, alongside the maturation of functional genomics tools such as RNA interference (RNAi), CRISPR-based genome editing, and transgenic approaches 2. These technologies have enabled systematic dissection of P450 function across diverse insect taxa and ecological contexts. Meanwhile, the development of heterologous expression systems has facilitated biochemical characterization of P450 enzymes and their roles in xenobiotic metabolism 2. More recently, dedicated resources, including Insect-eP450DB 194, the Insect Cytochrome P450 Database 195, and the Arthropod P450 Enchiridion 196, have further accelerated discovery by integrating genomic, functional, and evolutionary data. These advances provide an increasingly comprehensive foundation for understanding how P450 diversification underpins insect chemical adaptation. Although insect genome sequencing has advanced significantly, many assemblies remain incomplete, particularly in repetitive regions such as centromeres and telomeres. As sequencing technologies improve toward near-complete genome assemblies, additional and potentially lineage-specific P450 genes and novel CYPomes, are likely to be discovered, which may further reveal functional diversification in detoxification pathways.

In future studies, artificial intelligence (AI) is expected to become an important tool in future P450 research 197. Advances in AI-driven protein structure prediction and protein-ligand modeling may facilitate the identification of substrate recognition features, prediction of metabolic capabilities, and functional characterization of newly discovered P450 enzymes. In addition, AI-guided modeling may support the rational design of synergists targeting key detoxification P450s and contribute to the development of host-microbe co-metabolism frameworks for a more integrated understanding of xenobiotic detoxification. Future studies should also integrate structural biology, genome-editing technologies such as CRISPR/Cas, and applied pest management strategies to bridge molecular mechanisms with functional validation and practical applications 198. Such multidisciplinary approaches will be essential for validating P450 function *in vivo*, elucidating the evolutionary dynamics of insecticide resistance, and translating mechanistic insights into actionable outcomes. These may include the development of novel synergists targeting key detoxification enzymes, as well as microbiome-informed and systems-based strategies for more sustainable pest and pollinator management.

## Supplementary Material

Supplementary figure and table.

## Abbreviations

ABC transporters: ATP-binding cassette transporters
CHC: cuticular hydrocarbons
CncC: cap “n” collar isoform-C
CNV: copy number variance
COEs: carboxylesterases
EDMP: 2-ethyl-3,6(5)-dimethylpyrazine
GPCR: G protein-coupled receptor
GSTs: glutathione S-transferases
HIPVs: herbivore-induced plant volatiles
P450: Cytochrome P450 monooxygenase
RNAi: RNA interference
ROS: reactive oxygen species
SRS: substrate recognition site
UGTs: UDP-glycosyltransferases
