# Supplementary material for: Complex Roles of Insect Cytochrome P450s in Chemical Adaptation
Source: Int J Biol Sci. 2026 Jun 10;22(12):6281–97. doi: 10.7150/ijbs.135523 (PMC13411725; doi:10.7150/ijbs.135523)
Supplement: Supplementary file 1 — Supplementary figure and table. [file ijbsv22p6281s1.pdf]

Complex Roles of Insect Cytochrome P450s in Chemical Adaptation

Qi-Ren Chen[1], Timothy W. Moural[1], Fang Zhu[1,2]

Supplementary data:

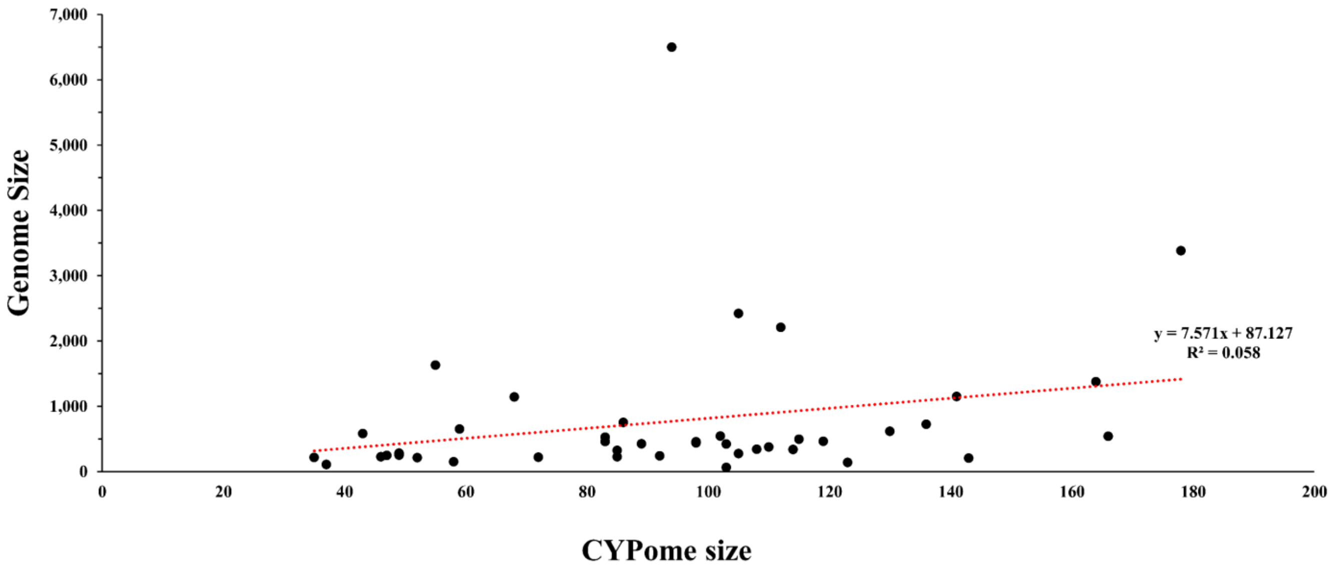

Figure S1. Lack of correlation between genome size and cytochrome P450 (CYP) gene number across insect species. Data are derived from 50 species with genome information available listed in Table S1, excluding transcriptome-based datasets. Each point represents one species, and the dashed line denotes the linear regression fit, illustrating that variation in CYPome size is largely decoupled from genome size.

Table S1. Numbers of CYP families and genes in each clan across diverse insect species.

Order	Scientific Name	Genome size (Mb)	Clan 2	Mito Clan	Clan 3	Clan 4	Total	Reference
Coleoptera (6)	*Brassicogethes aeneus*	62	7	9	50	37	**103**	King *et al.*, 2023 [1] (Table S7)
	Dendroctonus ponderosae	225	7	9	47	22	**85**	Keeling *et al.*, 2013[2]; Keeling *et al.*, 2022 [3]
	Diabrotica virgifera virgifera	2,420	9	13	58	25	**105**	Coates *et al.*, 2023 [4] (Fig. 4)
	Harmonia axyridis	423	10	9	42	28	**89**	Chen *et al.*, 2021 [5]
	Leptinotarsa decemlineata	--	2	7	61	28	**98**	Zhu *et al.*, 2016 [6] (Transcriptomes)
	Tribolium castenaum	204	8	9	79	47	**143**	Zhu *et al.*, 2013[7]; Richards *et al.*, 2008 [8]
Subaverage**			8	10	55	32	105	
Stdv**			1	2	15	10	23	
Diptera (11)	*Aedes aegypti*	1,376	11	10	84	59	**164**	Reddy *et al.*, 2012 [9] (Table 2a); Nene *et al.*, 2007 [10]
	Anopheles gambiae	273	10	9	40	46	**105**	Strode *et al.*, 2008[11]; Nene *et al.*, 2007 [10]
	Anopheles sinensis	2,208	9	9	53	41	**112**	Yan *et al.*, 2018[12]; Zhou *et al.*, 2014 [13]
	Bactrocera dorsalis	542	7	15	45	35	**102**	Jiang *et al.*, 2022 [14] (Table S4 and S13)
	Bactrocera latifrons	463	9	22	49	39	**119**	Jiang *et al.*, 2022 [14] (Table S4 and S13)
	Bactrocera oleae	455	8	18	44	28	**98**	Jiang *et al.*, 2022 [14] (Table S13); Hansen *et al.*, 2026 [15]
	Ceratitis capitata	436	7	17	51	23	**98**	Jiang *et al.*, 2022 [14] (Table S4 and S13)
	Culex quinquefasciatus	540	13	10	77	66	**166**	Reddy *et al.*, 2012 [9] (Table 2a); Zhou *et al.*, 2014 [13]
	Drosophila melanogaster	138	11	19	54	39	**123**	Jiang *et al.*, 2022 [14] (Table S4 and S13)
	Musca domestica	750	4	13	39	30	**86**	Li *et al.*, 2023[16]; Jiang *et al.*, 2022 [14] (Table S4)
	Zeugodacus cucurbitae	375	7	21	43	39	**110**	Jiang *et al.*, 2022 [14] (Table S4 and S13)
Subaverage**			9	15	53	41	117	
Stdv**			3	5	15	13	26	
Hemiptera (12)	*Acyrthosiphon pisum*	526	10	8	33	32	**83**	Ramsey *et al.*, 2010[17]; Mathers *et al.*, 2021 [18]
	Arma custos	--	5	7	48	31	**91**	Li *et al.*, 2023 [19] (Transcriptome)
	Bemisia tabaci MEAM1	615	10	7	57	56	**130**	Chen *et al.*, 2016 [20] (Table S13);
	Cimex lectularius	650	6	6	36	11	**59**	Benoit *et al.*, 2016 [21] (Table S22)
	Cyrtorhinus lividipennis	--	5	4	27	21	**57**	Li *et al.*, 2023 [19] (Transcriptome)

	Halyomorpha halys	1,150	6	6	84	45	**141**	Sparks *et al.*, 2020 [22]
	Murgantia histrionica	--	7	6	43	30	**86**	Li *et al.*, 2023 [19] (Transcriptome)
	Nilaparvata lugens	1,140	10	12	19	27	**68**	Lao *et al.*, 2015[23]; Xue *et al.*, 2014 [24]
	Orius laevigatus	151	6	5	34	13	**58**	Bailey *et al.*, 2022 [25]
	Pseudoregma bambucicola	582	6	7	13	17	**43**	Lu *et al.*, 2023 [26] (Table S2); Zhang *et al.*, 2024 [27]
	Rhodnius prolixus	--	7	8	55	49	**119**	Li *et al.*, 2023 [19] (Transcriptome)
	Triatoma infestans	--	1	6	65	22	**94**	Li *et al.*, 2023 [19] (Transcriptome)
Subaverage**			8	7	39	29	83	
Stdv**			2	2	24	17	38	
Hymenoptera (8)	*Apis mellifera*	224	8	6	28	4	**46**	Claudianos *et al.*, 2006 [28] (Table 1); Wallberg *et al.*, 2019 [29]
	Apis florea	214	6	5	21	3	**35**	Beadle *et al.*, 2019 [30] (Table S5); Tan *et al.*, 2021 [31]
	Bombus_impatiens	247	7	6	30	4	**47**	Beadle *et al.*, 2019 [30] (Table S5); Sadd *et al.*, 2015 [32]
	Bombus terrestris	249	7	6	32	4	**49**	Beadle *et al.*, 2019 [30] (Table S5); Sadd *et al.*, 2015 [32]
	Megachile rotundata	281	7	6	32	4	**49**	Hayward *et al.*, 2019 [33] (Table S1); Shi *et al.*, 2025 [34]
	Nasonia vitripennis	238	7	7	49	29	**92**	Sadd *et al.*, 2015 [32] (Table 3); Smith *et al.*, 2011 [35] (Table S3)
	Osmia bicornis	213	8	6	33	5	**52**	Beadle *et al.*, PLoS Genetics, 2019 [30] (Table S5);
	Pogonomyrmex Barbatus	220	7	7	40	18	**72**	Sadd *et al.*, 2015 [32] (Table 3); Smith *et al.*, 2011 [35] (Table S3)
Subaverage**			7	6	33	9	55	
Stdv**			1	1	8	9	18	
Lepidoptera (8)	*Bombyx mori*	460	7	11	31	34	**83**	Kawamoto *et al.*, 2019 [36]
	Cnaphalocrocis medinalis	--	5	6	16	9	**36**	Zhang *et al.*, 2018 [37] (Table 3, Transcriptome)
	Cydia pomonella	723	8	14	67	47	**136**	Wan *et al.*, 2019, (Table 14)
	Danaus plexippus	--	8	12	36	30	**86**	Zhang *et al.*, 2018 [37] (Table 3, Transcriptome)
	Helicoverpa armigera	337	8	10	46	50	**114**	Pearce *et al.*, 2017 [38] (Table 2)
	Helicoverpa zea	341	8	10	42	48	**108**	Pearce *et al.*, 2017 [38] (Table 2)
	Manduca sexta	419	8	16	45	34	**103**	Kanost *et al.*, 2016 [39] (Table S15)
	Plutella xylostella	323	10	13	26	36	**85**	Yu *et al.*, 2015[40]; Boyes *et al.*, 2023 [41] ; You *et al.*, 2013 [42]
Subaverage**			8	12	43	42	104	

Stdv**			1	2	14	8	20	
Blattodea (1)	*Periplaneta americana*	3,380	23	13	79	62	**178**	Li *et al.*, 2018 [43] (Fig. 2)
Odonata (1)	*Calopteryx splendens*	1,630	20	9	18	8	**55***	Ioannidis *et al.*, 2017 [44] (Table S1)
Orthoptera (1)	*Locusta migratoria*	6,500	9	9	54	21	**94**	Wang *et al.*, 2014 [45] (Fig. S31)
Psocodea (1)	*Pediculus humanus*	108	8	8	12	9	**37**	Lee *et al.*, 2010[46]; Kirkness *et al.* 2010 [47]
Siphonaptera (1)	*Ctenocephalides felis*	433-551	11	34	57	13	**115**	Feyereisen, *Curr. Res. Insect Sci.*, 2022[48]; Driscoll et al., *BMC Biology*, 2020[49]
Average (42)**			9	11	45	30	94	

* Do not include Clan 20; "--" represents Transcriptome. ** The transcriptome data was excluded from calculation.

Reference

(1) King, R.; Boaventura, D.; Hunt, B.J.; Hayward, A.; Gutbrod, O.; Williamson, M.S.; Bass, C.; Nauen, R. A chromosome-scale genome assembly of the pollen beetle, *Brassicogethes aeneus*, provides insight into cytochrome P450-mediated pyrethroid resistance. *Entomologia Generalis* **2023**, *43* (3), 639-648. DOI: 10.1127/entomologia/2023/1832.

(2) Keeling, C. I.; Yuen, M. M.; Liao, N. Y.; Docking, T. R.; Chan, S. K.; Taylor, G. A.; Palmquist, D. L.; Jackman, S. D.; Nguyen, A.; Li, M.; et al. Draft genome of the mountain pine beetle, *Dendroctonus ponderosae* Hopkins, a major forest pest. *Genome Biol* **2013**, *14* (3), R27. DOI: 10.1186/gb-2013-14-3-r27.

(3) Keeling, C. I.; Campbell, E. O.; Batista, P. D.; Shegelski, V. A.; Trevoy, S. A. L.; Huber, D. P. W.; Janes, J. K.; Sperling, F. A. H. Chromosome-level genome assembly reveals genomic architecture of northern range expansion in the mountain pine beetle, *Dendroctonus ponderosae* Hopkins (Coleoptera: Curculionidae). *Mol Ecol Resour* **2022**, *22* (3), 1149-1167. DOI: 10.1111/1755-0998.13528.

(4) Coates, B. S.; Walden, K. K. O.; Lata, D.; Vellichirammal, N. N.; Mitchell, R. F.; Andersson, M. N.; McKay, R.; Lorenzen, M. D.; Grubbs, N.; Wang, Y. H.; et al. A draft *Diabrotica virgifera virgifera* genome: insights into control and host plant adaption by a major maize pest insect. *BMC Genomics* **2023**, *24* (1), 19. DOI: 10.1186/s12864-022-08990-y.

(5) Chen, M.; Mei, Y.; Chen, X.; Xiao, D.; He, K.; Li, Q.; Wu, M.; Wang, S.; Zhang, F.; Li, F. A chromosome-level assembly of the harlequin ladybird *Harmonia axyridis* as a genomic resource to study beetle and invasion biology. *Mol Ecol Resour* **2021**, *21* (4), 1318-1332. DOI: 10.1111/1755-0998.13342.

(6) Zhu, F.; Moural, T. W.; Nelson, D. R.; Palli, S. R. A specialist herbivore pest adaptation to xenobiotics through up-regulation of multiple Cytochrome P450s. *Sci Rep* **2016**, *6*, 20421. DOI: 10.1038/srep20421.

(7) Zhu, F.; Moural, T. W.; Shah, K.; Palli, S. R. Integrated analysis of cytochrome P450 gene superfamily in the red flour beetle, *Tribolium castaneum*. *BMC Genomics* **2013**, *14*, 174. DOI: 10.1186/1471-2164-14-174.

(8) Richards, S.; Gibbs, R. A.; Weinstock, G. M.; Brown, S. J.; Denell, R.; Beeman, R. W.; Gibbs, R.; Bucher, G.; Friedrich, M.; Grimmelikhuijzen, C. J.; et al. The genome of the model beetle and pest *Tribolium castaneum*. *Nature* **2008**, *452* (7190), 949-955. DOI: 10.1038/nature06784.

(9) Reddy, B. N.; Rao, B. P.; Prasad, G.; Raghavendra, K. Identification and classification of detoxification enzymes from *Culex quinquefasciatus* (Diptera: Culicidae). *Bioinformation* **2012**, *8* (9), 430-436. DOI: 10.6026/97320630008430.
(10) Nene, V.; Wortman, J. R.; Lawson, D.; Haas, B.; Kodira, C.; Tu, Z. J.; Loftus, B.; Xi, Z.; Megy, K.; Grabherr, M.; et al. Genome sequence of *Aedes aegypti*, a major arbovirus vector. *Science* **2007**, *316* (5832), 1718-1723. DOI: 10.1126/science.1138878.
(11) Strode, C.; Wondji, C. S.; David, J. P.; Hawkes, N. J.; Lumjuan, N.; Nelson, D. R.; Drane, D. R.; Karunaratne, S. H.; Hemingway, J.; Black, W. C.; et al. Genomic analysis of detoxification genes in the mosquito *Aedes aegypti*. *Insect Biochem Mol Biol* **2008**, *38* (1), 113-123. DOI: 10.1016/j.ibmb.2007.09.007.
(12) Yan, Z. W.; He, Z. B.; Yan, Z. T.; Si, F. L.; Zhou, Y.; Chen, B. Genome-wide and expression-profiling analyses suggest the main cytochrome P450 genes related to pyrethroid resistance in the malaria vector, Anopheles sinensis (Diptera Culicidae). *Pest Manag Sci* **2018**, *74* (8), 1810-1820. DOI: 10.1002/ps.4879.
(13) Zhou, D.; Zhang, D.; Ding, G.; Shi, L.; Hou, Q.; Ye, Y.; Xu, Y.; Zhou, H.; Xiong, C.; Li, S.; et al. Genome sequence of *Anopheles sinensis* provides insight into genetics basis of mosquito competence for malaria parasites. *BMC Genomics* **2014**, *15*, 42. DOI: 10.1186/1471-2164-15-42.
(14) Jiang, F.; Liang, L.; Wang, J.; Zhu, S. Chromosome-level genome assembly of *Bactrocera dorsalis* reveals its adaptation and invasion mechanisms. *Commun Biol* **2022**, *5* (1), 25. DOI: 10.1038/s42003-021-02966-6.
(15) Hansen, T. E.; Corpuz, R. L.; Simmonds, T. J.; Aldebron, C.; Mason, C. J.; Geib, S. M.; Sim, S. B. Genome report: chromosome-scale genome assembly of the olive fly *Bactrocera oleae* (Diptera: Tephritidae). *G3 (Bethesda)* **2026**, *16* (1). DOI: 10.1093/g3journal/jkaf235.
(16) Li, M.; Feng, X.; Reid, W. R.; Tang, F.; Liu, N. Multiple-P450 gene co-up-regulation in the development of permethrin resistance in the house fly. *Int J Mol Sci* **2023**, *24* (4). DOI: 10.3390/ijms24043170.
(17) Ramsey, J. S.; Rider, D. S.; Walsh, T. K.; De Vos, M.; Gordon, K. H.; Ponnala, L.; Macmil, S. L.; Roe, B. A.; Jander, G. Comparative analysis of detoxification enzymes in *Acyrthosiphon pisum* and *Myzus persicae*. *Insect Mol Biol* **2010**, *19 Suppl 2*, 155-164. DOI: 10.1111/j.1365-2583.2009.00973.x.
(18) Mathers, T. C.; Wouters, R. H. M.; Mugford, S. T.; Swarbreck, D.; van Oosterhout, C.; Hogenhout, S. A. Chromosome-scale genome assemblies of aphids reveal extensively rearranged autosomes and long-term conservation of the X chromosome. *Mol Biol Evol* **2021**, *38* (3), 856-875. DOI: 10.1093/molbev/msaa246.
(19) Li, W.; Zou, J.; Yang, X.; Yang, M.; Jiang, P.; Wang, X.; Huang, C.; He, Y. Identification of metabolizing enzyme genes associated with xenobiotics and odorants in the predatory stink bug. *Heliyon* **2023**, *9* (8), e18657. DOI: 10.1016/j.heliyon.2023.e18657.
(20) Chen, W.; Hasegawa, D. K.; Kaur, N.; Kliot, A.; Pinheiro, P. V.; Luan, J.; Stensmyr, M. C.; Zheng, Y.; Liu, W.; Sun, H.; et al. The draft genome of whitefly Bemisia tabaci MEAM1, a global crop pest, provides novel insights into virus transmission, host adaptation, and insecticide resistance. *BMC Biol* **2016**, *14* (1), 110. DOI: 10.1186/s12915-016-0321-y.
(21) Benoit, J. B.; Adelman, Z. N.; Reinhardt, K.; Dolan, A.; Poelchau, M.; Jennings, E. C.; Szuter, E. M.; Hagan, R. W.; Gujar, H.; Shukla, J. N.; et al. Unique features of a global human ectoparasite identified through sequencing of the bed bug genome. *Nat Commun* **2016**, *7*, 10165. DOI: 10.1038/ncomms10165.
(22) Sparks, M. E.; Bansal, R.; Benoit, J. B.; Blackburn, M. B.; Chao, H.; Chen, M.; Cheng, S.; Childers, C.; Dinh, H.; Doddapaneni, H. V.; et al. Brown marmorated stink bug, *Halyomorpha halys* (Stål), genome: putative underpinnings of polyphagy, insecticide resistance potential and biology of a top worldwide pest. *BMC Genomics* **2020**, *21* (1), 227. DOI: 10.1186/s12864-020-6510-7.

(23) Lao, S. H.; Huang, X. H.; Huang, H. J.; Liu, C. W.; Zhang, C. X.; Bao, Y. Y. Genomic and transcriptomic insights into the cytochrome P450 monooxygenase gene repertoire in the rice pest brown planthopper, *Nilaparvata lugens*. *Genomics* **2015**, *106* (5), 301-309. DOI: 10.1016/j.ygeno.2015.07.010.
(24) Xue, J.; Zhou, X.; Zhang, C. X.; Yu, L. L.; Fan, H. W.; Wang, Z.; Xu, H. J.; Xi, Y.; Zhu, Z. R.; Zhou, W. W.; et al. Genomes of the rice pest brown planthopper and its endosymbionts reveal complex complementary contributions for host adaptation. *Genome Biol* **2014**, *15* (12), 521. DOI: 10.1186/s13059-014-0521-0.
(25) Bailey, E.; Field, L.; Rawlings, C.; King, R.; Mohareb, F.; Pak, K. H.; Hughes, D.; Williamson, M.; Ganko, E.; Buer, B.; et al. A scaffold-level genome assembly of a minute pirate bug, *Orius laevigatus* (Hemiptera: Anthocoridae), and a comparative analysis of insecticide resistance-related gene families with hemipteran crop pests. *BMC Genomics* **2022**, *23* (1), 45. DOI: 10.1186/s12864-021-08249-y.
(26) Lu, J.; Zhang, H.; Wang, Q.; Huang, X. Genome-wide identification and expression pattern of cytochrome P450 genes in the social aphid. *Insects* **2023**, *14* (2). DOI: 10.3390/insects14020212.
(27) Zhang, H.; Liu, Q.; Lu, J.; Wu, L.; Cheng, Z.; Qiao, G.; Huang, X. Genomic and transcriptomic analyses of a social hemipteran provide new insights into insect sociality. *Mol Ecol Resour* **2024**, *24* (8), e14019. DOI: 10.1111/1755-0998.14019.
(28) Claudianos, C.; Ranson, H.; Johnson, R. M.; Biswas, S.; Schuler, M. A.; Berenbaum, M. R.; Feyereisen, R.; Oakeshott, J. G. A deficit of detoxification enzymes: pesticide sensitivity and environmental response in the honeybee. *Insect Mol Biol* **2006**, *15* (5), 615-636. DOI: 10.1111/j.1365-2583.2006.00672.x.
(29) Wallberg, A.; Bunikis, I.; Pettersson, O. V.; Mosbech, M. B.; Childers, A. K.; Evans, J. D.; Mikheyev, A. S.; Robertson, H. M.; Robinson, G. E.; Webster, M. T. A hybrid de novo genome assembly of the honeybee, *Apis mellifera*, with chromosome-length scaffolds. *BMC Genomics* **2019**, *20* (1), 275. DOI: 10.1186/s12864-019-5642-0.
(30) Beadle, K.; Singh, K. S.; Troczka, B. J.; Randall, E.; Zaworra, M.; Zimmer, C. T.; Hayward, A.; Reid, R.; Kor, L.; Kohler, M.; et al. Genomic insights into neonicotinoid sensitivity in the solitary bee *Osmia bicornis*. *PLoS Genet* **2019**, *15* (2), e1007903. DOI: 10.1371/journal.pgen.1007903.
(31) Tan, H.; Naeem, M.; Ali, H.; Shakeel, M.; Kuang, H.; Zhang, Z.; Sun, C. Genome sequence of the Asian honeybee in Pakistan sheds light on its phylogenetic relationship with other honeybees. *Insects* **2021**, *12* (7). DOI: 10.3390/insects12070652.
(32) Sadd, B. M.; Barribeau, S. M.; Bloch, G.; de Graaf, D. C.; Dearden, P.; Elsik, C. G.; Gadau, J.; Grimmelikhuijzen, C. J.; Hasselmann, M.; Lozier, J. D.; et al. The genomes of two key bumblebee species with primitive eusocial organization. *Genome Biol* **2015**, *16* (1), 76. DOI: 10.1186/s13059-015-0623-3.
(33) Hayward, A.; Beadle, K.; Singh, K. S.; Exeler, N.; Zaworra, M.; Almanza, M. T.; Nikolakis, A.; Garside, C.; Glaubitz, J.; Bass, C.; et al. The leafcutter bee, *Megachile rotundata*, is more sensitive to N-cyanoamidine neonicotinoid and butenolide insecticides than other managed bees. *Nat Ecol Evol* **2019**, *3* (11), 1521-1524. DOI: 10.1038/s41559-019-1011-2.
(34) Shi, R.; Duan, P.; Zhu, M.; Zhang, R.; Zhao, Z.; Nie, X.; He, H.; Hou, L.; Wang, X. Chromosome-level genome assembly of the leafcutter bee *Megachile rotundata* reveals its ecological adaptation and pollination biology. *Adv Sci* **2025**, e2417054. DOI: 10.1002/advs.202417054.
(35) Smith, C. R.; Smith, C. D.; Robertson, H. M.; Helmkampf, M.; Zimin, A.; Yandell, M.; Holt, C.; Hu, H.; Abouheif, E.; Benton, R.; et al. Draft genome of the red harvester ant *Pogonomyrmex barbatus*. *Proc Natl Acad Sci U S A* **2011**, *108* (14), 5667-5672. DOI: 10.1073/pnas.1007901108.
(36) Kawamoto, M.; Jouraku, A.; Toyoda, A.; Yokoi, K.; Minakuchi, Y.; Katsuma, S.; Fujiyama, A.; Kiuchi, T.; Yamamoto, K.; Shimada, T. High-quality genome assembly of the silkworm, *Bombyx mori*. *Insect Biochem Mol Biol* **2019**, *107*, 53-62. DOI: 10.1016/j.ibmb.2019.02.002.

(37) Zhang, H.; Zhao, M.; Liu, Y.; Zhou, Z.; Guo, J. Identification of cytochrome P450 monooxygenase genes and their expression in response to high temperature in the alligatorweed flea beetle *Agasicles hygrophila* (Coleoptera: Chrysomelidae). *Sci Rep* **2018**, *8* (1), 17847. DOI: 10.1038/s41598-018-35993-1.
(38) Pearce, S. L.; Clarke, D. F.; East, P. D.; Elfekih, S.; Gordon, K. H. J.; Jermiin, L. S.; McGaughran, A.; Oakeshott, J. G.; Papanicolaou, A.; Perera, O. P.; et al. Genomic innovations, transcriptional plasticity and gene loss underlying the evolution and divergence of two highly polyphagous and invasive *Helicoverpa* pest species. *BMC Biol* **2017**, *15* (1), 63. DOI: 10.1186/s12915-017-0402-6.
(39) Kanost, M. R.; Arrese, E. L.; Cao, X.; Chen, Y. R.; Chellapilla, S.; Goldsmith, M. R.; Grosse-Wilde, E.; Heckel, D. G.; Herndon, N.; Jiang, H.; et al. Multifaceted biological insights from a draft genome sequence of the tobacco hornworm moth, *Manduca sexta*. *Insect Biochem Mol Biol* **2016**, *76*, 118-147. DOI: 10.1016/j.ibmb.2016.07.005.
(40) Yu, L.; Tang, W.; He, W.; Ma, X.; Vasseur, L.; Baxter, S. W.; Yang, G.; Huang, S.; Song, F.; You, M. Characterization and expression of the cytochrome P450 gene family in diamondback moth, *Plutella xylostella* (L.). *Sci Rep* **2015**, *5*, 8952. DOI: 10.1038/srep08952.
(41) Boyes, D.; Lab, U. o. O. a. W. W. G. A.; collective, D. T. o. L. B.; programme, W. S. I. T. o. L.; collective, W. S. I. S. O. D. P.; collective, T. o. L. C. I.; Consortium, D. T. o. L. The genome sequence of the diamondback moth,. *Wellcome Open Res* **2023**, *8*, 404. DOI: 10.12688/wellcomeopenres.20006.1.
(42) You, M.; Yue, Z.; He, W.; Yang, X.; Yang, G.; Xie, M.; Zhan, D.; Baxter, S. W.; Vasseur, L.; Gurr, G. M.; et al. A heterozygous moth genome provides insights into herbivory and detoxification. *Nat Genet* **2013**, *45* (2), 220-225. DOI: 10.1038/ng.2524.
(43) Li, S.; Zhu, S.; Jia, Q.; Yuan, D.; Ren, C.; Li, K.; Liu, S.; Cui, Y.; Zhao, H.; Cao, Y.; et al. The genomic and functional landscapes of developmental plasticity in the American cockroach. *Nat Commun* **2018**, *9* (1), 1008. DOI: 10.1038/s41467-018-03281-1.
(44) Ioannidis, P.; Simao, F. A.; Waterhouse, R. M.; Manni, M.; Seppey, M.; Robertson, H. M.; Misof, B.; Niehuis, O.; Zdobnov, E. M. Genomic Features of the Damselfly Calopteryx splendens Representing a Sister Clade to Most Insect Orders. *Genome Biol Evol* **2017**, *9* (2), 415-430. DOI: 10.1093/gbe/evx006.
(45) Wang, X.; Fang, X.; Yang, P.; Jiang, X.; Jiang, F.; Zhao, D.; Li, B.; Cui, F.; Wei, J.; Ma, C.; et al. The locust genome provides insight into swarm formation and long-distance flight. *Nat Commun* **2014**, *5*, 2957. DOI: 10.1038/ncomms3957.
(46) Lee, S. H.; Kang, J. S.; Min, J. S.; Yoon, K. S.; Strycharz, J. P.; Johnson, R.; Mittapalli, O.; Margam, V. M.; Sun, W.; Li, H. M.; et al. Decreased detoxification genes and genome size make the human body louse an efficient model to study xenobiotic metabolism. *Insect Mol Biol* **2010**, *19* (5), 599-615. DOI: 10.1111/j.1365-2583.2010.01024.x.
(47) Kirkness, E. F.; Haas, B. J.; Sun, W.; Braig, H. R.; Perotti, M. A.; Clark, J. M.; Lee, S. H.; Robertson, H. M.; Kennedy, R. C.; Elhaik, E.; et al. Genome sequences of the human body louse and its primary endosymbiont provide insights into the permanent parasitic lifestyle. *Proc Natl Acad Sci U S A* **2010**, *107* (27), 12168-12173. DOI: 10.1073/pnas.1003379107.
(48) Feyereisen, R. The P450 genes of the cat flea,. *Curr Res Insect Sci* **2022**, *2*, 100032. DOI: 10.1016/j.cris.2022.100032.
(49) Driscoll, T. P.; Verhoeve, V. I.; Gillespie, J. J.; Johnston, J. S.; Guillotte, M. L.; Rennoll-Bankert, K. E.; Rahman, M. S.; Hagen, D.; Elsik, C. G.; Macaluso, K. R.; et al. A chromosome-level assembly of the cat flea genome uncovers rampant gene duplication and genome size plasticity. *BMC Biol* **2020**, *18* (1), 70. DOI: 10.1186/s12915-020-00802-7.
